# Influence of Body Mass Index (BMI) and Waist–Hip Ratio (WHR) on Selected Semen Parameters

**DOI:** 10.3390/ijms26094089

**Published:** 2025-04-25

**Authors:** Kamil Gill, Monika Fraczek, Maciej Kurpisz, Małgorzata Piasecka

**Affiliations:** 1 Department of Histology and Developmental Biology, Faculty of Health Sciences, Pomeranian Medical University in Szczecin, 71-210 Szczecin, Poland; 2 Institute of Human Genetics, Polish Academy of Sciences, 60-479 Poznan, Poland; monika.fraczek@igcz.poznan.pl (M.F.); maciej.kurpisz@igcz.poznan.pl (M.K.)

**Keywords:** male obesity, oxidative stress antioxidant capacity, hyaluronan-binding assay, sperm DNA integrity

## Abstract

Because male obesity may result in reproductive failure, we aimed to examine the possible links among body mass index (BMI), the waist–hip ratio (WHR), and basic semen parameters, the oxidation–reduction potential of semen, the total antioxidant capacity of seminal plasma, the ability of sperm to bind hyaluronic acid, and sperm DNA fragmentation (SDF). This study was performed on semen (*n* = 543) collected from volunteers classified as follows: normal weight (BMI 18.5–24.9 kg/m^2^), overweight (BMI 25.0–29.9 kg/m^2^), obese (BMI ≥ 30.0 kg/m^2^), with a normal WHR (<1) or abnormal WHR (≥1). No significant differences in standard semen parameters were found between men with a normal BMI and those with overweight/obesity. However, compared with overweight men, obese men had a higher SDF index prevalence and risk for an SDF index > 20%. Compared with men with WHR < 1, those with WHR ≥ 1 had significantly lower sperm motility, morphology, and vitality and an increased SDF index, prevalence and risk for an SDF index > 20%. In conclusion, abnormal WHR had a greater negative impact on conventional semen parameters than abnormal BMI. Both BMI ≥ 30.0 and WHR ≥ 1 negatively influenced sperm chromatin integrity only. Obesity is a potential risk factor for sperm DNA damage.

## 1. Introduction

In recent years, male infertility has been recognized as a global public health concern. Currently, this is a common medical problem requiring scientific advances in diagnostics and personalized medical approaches. Globally, male factors are estimated to contribute to approximately 50% of cases of childlessness. In 20% of cases, it is an isolated factor, and in 30% of cases, it coexists with female factors [1,2]. The etiology of male infertility is complex and multifactorial. The causes and risk factors for male infertility include congenital factors (e.g., anorchia, cryptorchidism, Y chromosome microdeletion, chromosomal or genetic abnormalities, genetic endocrinopathy), acquired factors (e.g., varicocele, testicular trauma, torsion, germ cell tumors, acquired hypogonadotropic hypogonadism, urogenital infections, chemotherapy, medications, radiation, heat, systemic diseases) and idiopathic risk factors (e.g., environmental or occupational exposure to toxins, smoking, alcohol, recreational drugs, dietary factors, obesity, advanced paternal age) [3,4,5,6].

Notably, in the current literature on male infertility, special attention is given to the relationship between obesity and male infertility/subfertility [7,8,9,10,11,12,13,14,15,16,17]. Obesity, defined as a body mass index (BMI) of ≥30 kg/m^2^, is a growing epidemic, particularly in highly developed countries, and is characterized by excessive and abnormal accumulation of adipose tissue (very often dysfunctional). According to data provided by the World Health Organization (WHO), in 2022, 43% of adults (aged ≥ 18 years) were overweight (BMI ≥ 25.0–29.9), whereas 16% were obese. Moreover, the number of adults with obesity doubled between 1990 and 2022 (https://www.who.int/news-room/fact-sheets/detail/obesity-and-overweight (accessed on 25 February 2025)). Importantly, obesity is a common condition and a major cause of morbidity and mortality [15,18,19].

Obesity affects an increasing number of men of reproductive age. It is a chronic disease accompanied by metabolic derangements and various comorbidities (i.e., insulin resistance, diabetes, microvascular disease, hypertension, heat stress, nonalcoholic fatty liver disease, obstructive sleep apnea), and endocrine abnormalities result from alterations in the hypothalamic–pituitary–testis axis, which may consequently lead to gonadal dysfunction at the level of steroidogenesis and spermatogenesis. Moreover, obesity is associated with the generation of proinflammatory cytokines and pathological amounts of reactive oxygen species (ROS) as a result of oxidative stress, which also has a negative effect on the function of the male gonad [9,12,15,16,17,19,20,21,22,23]. Eventually, hypogonadism, a decrease in basic semen parameters, abnormal sperm chromatin maturity and integrity, and epigenetic changes in male gametes (e.g., DNA methylation, histone modification, and noncoding RNA alternation) may occur [7,8,13,16,17,19,20,21,22,23,24,25,26,27,28,29]. Both abnormal sperm chromatin status and epigenetic reprogramming can reduce the likelihood of conception, can negatively affect embryogenesis and can result in abnormal offspring phenotypes [30,31,32,33,34].

**Figure 1:**
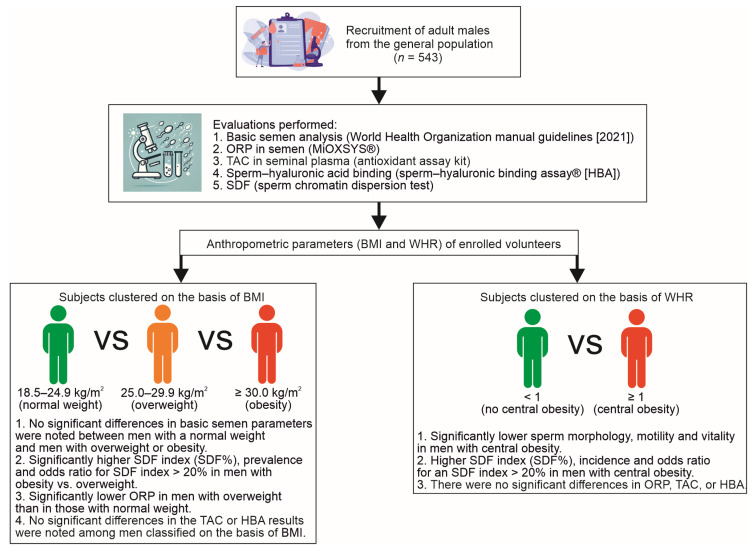
Research strategy.

Because the opinions of researchers regarding the relationship between obesity and the reproductive health of men are not always consistent [26,35], we decided to identify a potential link between body mass index (BMI) and waist–hip ratio (WHR) and standard sperm quality, oxidation–reduction potential (ORP) in semen, total antioxidant capacity (TAC) in seminal plasma, the ability of sperm to bind hyaluronic acid and sperm DNA fragmentation (SDF). To address these objectives, our study employed a strategy based on the comprehensive comparison of standard and nonstandard semen parameters in a general population of adult men, categorized according to their BMI (normal weight, overweight, or obesity) and WHR (without or with central obesity) (Figure 1).

## 2. Results

### 2.1. Study Parameters in BMI-Dependent Groups

A comparison of subjects clustered on the basis of BMI revealed that men with a BMI ranging from 18.5 to 24.9 kg/m^2^ (normal weight) were significantly younger (median: 32.00 y.o.) and had a lower BMI (median: 23.30 kg/m^2^) and WHR (median: 0.94) than men with a BMI ranging from 25 to 29.9 kg/m^2^ (overweight) (median: 33.00 y.o.; 26.89 kg/m^2^ and 0.99, respectively) and men with a BMI ≥ 30.0 kg/m^2^ (obesity) (median: 34.00 y.o.; 32.25 kg/m^2^ and 1.01, respectively). Additionally, significant differences in BMI (medians: 26.89 kg/m^2^ vs. 32.25 kg/m^2^) and WHR (median: 0.99 vs. 1.01) were noted between overweight and obese men (Table 1).

No significant differences (the Kruskal–Wallis test followed by Dunn’s multiple comparison test) in basic semen parameters were noted when the group of men with normal body weight was compared to the group of subjects with overweight and obesity, except for the percentage of nonprogressive sperm motility, which was lower in the first group than in the latter group (median: 6.00% vs. 7.00%). A comparison of two groups of men with abnormal BMI (overweight vs. obesity) revealed that the group with a BMI of 25.0–29.9 kg/m^2^ had a significantly greater percentage of sperm cells with progressive motility (median: 54.00% vs. 48.50%) and a lower percentage of nonprogressive sperm cells (median: 5.00% vs. 7.00%) (Table 2).

**Figure 2:**
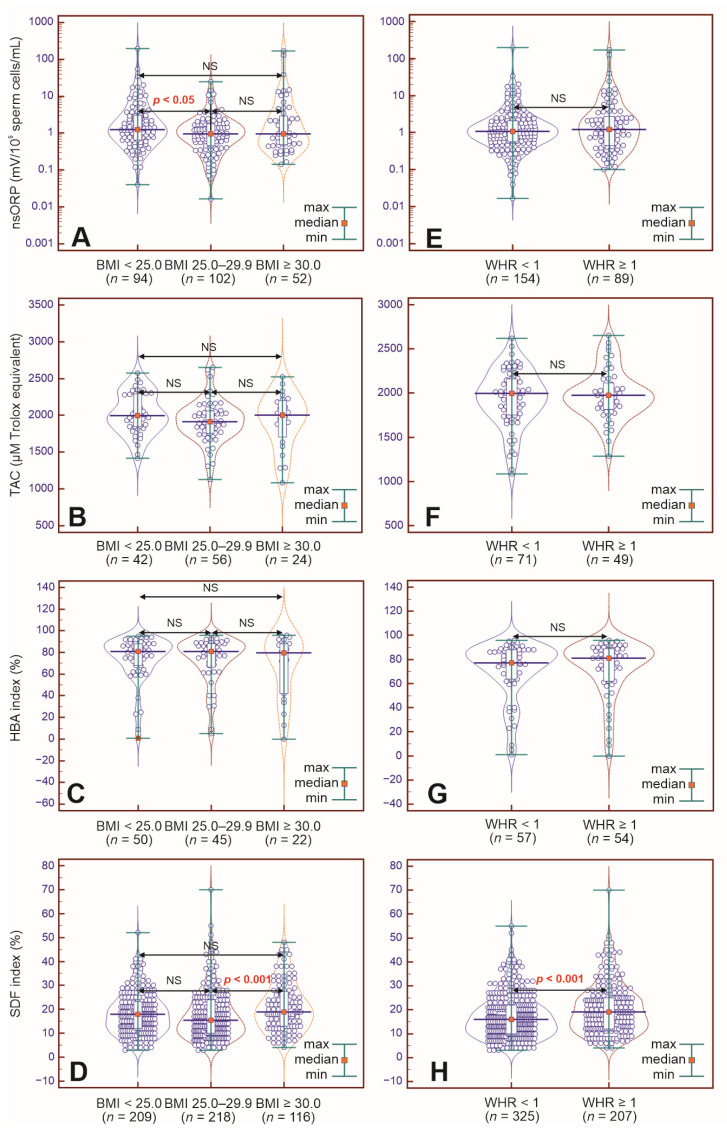
(**A**–**H**). Violin plots showing comparisons of nsORP (**A**,**E**), TAC (**B**,**F**), HBA (**C**,**G**), and SDF (**D**,**H**) results in BMI-dependent groups of men ((**A**–**D**); Kruskal–Wallis test followed by Dunn’s multiple comparison test) as well as with WHR-dependent groups ((**E**–**H**); Mann–Whitney U test). HBA—hyaluronic acid-binding assay, SDF—sperm DNA fragmentation, *n*—number of subjects, nsORP—normalized static oxidative–reduction potential, TAC—total antioxidant capacity. Statistical significance in both tests was noted when *p* < 0.05.

Additionally, regarding the normalized static oxidative–reduction potential in semen (nsORP), TAC, percentage of sperm cells binding to the hyaluronan layer (HBA index), and SDF index (nonstandard semen parameters), only a few differences were found. Of note, nsORP (mV/10^6^ sperm cells/mL) was significantly higher in the group of men with BMI < 25.0 kg/m^2^ than in the group of men with BMI 25.0–29.9 kg/m^2^ (median: 1.26 vs. 0.96), whereas the percentage of sperm cells with fragmented DNA (SDF index) was significantly lower in the group with BMI 25.0–29.9 kg/m^2^ than in the group with BMI ≥ 30.0 kg/m^2^ (median: 15.50% vs. 19.00%) (Figure 1 and Figure 2A–D). Moreover, the prevalence of subjects with nsORP > 1.37, TAC ≤ 1950 (µM Trolox equivalent), HBA index < 80%, and SDF index > 20% was estimated in each of the BMI-dependent groups. Chi^2^ test results revealed that there were significantly more men with an SDF index > 20% in the group of men with obesity than in the group of men with overweight (45.68% of participants vs. 30.73% of participants), and the odds ratio (OR) for an SDF index > 20% was almost 2-fold greater in the group of men with a BMI ≥ 30.0 kg/m^2^ than in the group with a BMI ranging from 25.0 to 29.9 kg/m^2^ (Figure 1, Table 3). No significant differences in terms of TAC, HBA index or SDF index results were noted between subjects with a normal BMI and those with overweight. In addition, no significant differences in nsORP, TAC, HBA index, or SDF index were noted between those with a normal BMI and those with obesity. Similarly, significant differences in the nsORP, TAC and HBA indices were not observed between the two groups with abnormal BMIs (Figure 1 and Figure 2A–D, Table 3).

### 2.2. Study Parameters in WHR-Dependent Groups

In the next stage of the statistical analysis, the men included in the study were divided into two groups: those with a WHR < 1 and those with a WHR ≥ 1 (central obesity). The Mann–Whitney U test confirmed that men with WHR values less than 1 were significantly younger (median: 32.00 vs. 34.00 y.o.), had a lower BMI (median: 25.00 kg/m^2^ vs. 27.90 kg/m^2^), and had a lower WHR (median: 0.94 vs. 1.02) (Table 4). Significant differences in conventional semen parameters were also noted. The group of men with a WHR < 1 had a greater percentage of sperm cells with a normal morphology (median: 2.00% vs. 1.00%), increased progressive motility (median: 55.00% vs. 48.00%), increased total motility (median: 63.00% vs. 55.00%), and greater proportions of live, eosin-negative and HOS test-positive cells (medians: 78.00% vs. 74.00% and 76.00% vs. 72.00%, respectively). Moreover, in the first group, the percentage of total sperm head defects was significantly lower (median: 96.00% vs. 98.00%) (Table 5).

A comparison of the nsORP, TAC, HBA index, and SDF index values revealed a significantly lower percentage of sperm cells with fragmented DNA in the group of men with WHR < 1 (median: 16.00% vs. 19.00%). No significant differences in the other parameters were observed (Figure 1 and Figure 2E–H). These results were confirmed by the estimation and comparison of the prevalence (Chi^2^ test) of the participants with nsORP > 1.37 (mV/10^6^ sperm cells/mL), TAC ≤ 1950 (µM Trolox equivalent), HBA index < 80% and SDF index > 20%. In the group with WHR in the normal range, there were significantly fewer men with > 20% sperm cells with fragmented DNA (30.76% of subjects vs. 44.44% of subjects). Moreover, men with abdominal (central) obesity had an almost 2-fold greater OR for an SDF index > 20% (Figure 1, Table 6).

## 3. Discussion

### 3.1. Limited Influence of BMI on Basic Semen Parameters

To achieve our goal, the study group of men was categorized into three groups on the basis of BMI: those with a normal BMI and those with a BMI indicating overweight or obesity (Figure 1). As expected, men with abnormal BMIs were significantly older than those in the first mentioned group were. Additionally, other authors confirmed that male aging is linked with an increase in BMI [36]. This phenomenon is well described in the available literature. Aging is associated with a decrease in the mass and size of internal organs and a decrease in the mass and volume of muscle tissue, with simultaneous increases in the amount of adipose tissue and the body mass weight. Sarcopenic obesity is occasionally diagnosed in elderly people (loss of skeletal muscle mass and function associated with obesity) [37,38,39]. Importantly, overweight and obesity are common medical conditions in infertile male patients. For example, Nikolic et al. [8] reported that in Western Europe, almost 9 out of 10 men in infertile couples had an abnormal (high) BMI. Therefore, we assumed that in our study, BMI-dependent groups would significantly differ in many standard semen parameters examined. However, unexpectedly, no significant differences in basic semen parameters, except nonprogressive motility, were observed between men with a normal BMI (18.5–24.9 kg/m^2^) and men with overweight (BMI 25.0–29.9 kg/m^2^) or with obesity (BMI ≥ 30.0 kg/m^2^). Only a comparison of men with overweight and men with obesity revealed a significantly lower percentage of sperm cells with progressive motility in the latter group. Similar results were described in a meta-analysis by Le et al. [26]. In this study, the authors found no effect of an abnormal BMI on the sperm concentration (other parameters were not analyzed). Moreover, the authors concluded that male overweight and obesity had no effect on in vitro fertilization outcomes, such as the clinical pregnancy rate and live birth rate. Additionally, Wang et al. [40] revealed that there were no differences in the prevalence of azoospermia (no sperm cells in the ejaculate), oligozoospermia (reduced sperm number), asthenozoospermia (reduced sperm motility) or teratozoospermia (reduced sperm morphology) between men with a normal BMI and those with overweight or obesity. Furthermore, using Spearman’s correlation coefficients, the authors did not find a significant linear (r_s_ ≥ 0.2) association between BMI and semen volume, sperm concentration, total number, sperm motility or morphology. A lack of significant differences in basic semen parameters (volume, pH, sperm number, motility and morphology) between BMI-dependent groups was also reported by Nikolic et al. [8]. Moreover, in this study, BMI was very weakly (r_s_ < 0.2) and insignificantly correlated with semen parameters as no significant linear relationship was noted between variables. On the other hand, Nikolic et al. [8] reported that BMI was negatively associated with embryo quality (in vitro fertilization treatment) but was not associated with the total number of obtained embryos or, more importantly, with clinical pregnancy rates. Additionally, a recent study [35] revealed that there were no significant differences in sperm number, motility or morphology or in the clinical pregnancy rate, abortion rate or live birth rate among patients with overweight and obesity and men with normal weight.

Nevertheless, some authors noted that a decline in semen quality (sperm concentration, morphology and motility) could be linked to an increase in the incidence of obesity in men over the past few decades with a concurrent decline in male fertility [9]. Notably, some studies presented mixed results. Eisenberg et al. [41] did not find a significant relationship between BMI and sperm concentration, motility, vitality or morphology; however, they reported that the prevalence of subjects with abnormal semen volume and abnormal sperm number increased with increasing BMI. Similarly, Dupont et al. [42] and Fariello et al. [43] reported no associations between BMI and total sperm count or sperm morphology; however, in these studies, sperm motility was significantly lower in men with obesity. Additionally, no obvious influence of BMI on semen parameters was noted by Tunc et al. [44]. The authors showed linear associations between BMI and sperm concentration but not with sperm morphology. In another original study, overweight and obesity were significantly associated with reduced standard semen parameters, mostly semen volume, total sperm number and total motile sperm count [10,11]. Additionally, in another study, Esmaeili et al. [12] reported that men with a normal BMI had significantly greater sperm morphology, motility and vitality than did those with overweight and obesity. Keszthelyi et al. [28] used regression models and reported that BMI was significantly correlated with all evaluated basic semen parameters.

### 3.2. Influence of WHR on Basic Semen Parameters

One of the most commonly used anthropometric parameters to verify the normality of body weight and the cornerstone of the current classification system for obesity is BMI [45]. However, BMI has several limitations. Therefore, we decided to use a complementary measurement, WHR, which is less subject to influence from height and muscle mass. Additionally, WHR is a simple and cost-effective method that can be used for the verification of central (abdominal) obesity [13,46]. Notably, the accumulation of excessive fat tissue in the abdomen has a particularly negative impact on health, including men’s reproductive health [13].

In our study, WHR-dependent groups, similar to BMI-dependent groups, significantly differed in age. The participants with high WHR (≥1) were older. These findings seem to confirm our previous conclusions that obesity is an age-dependent condition. Additionally, other authors [27] have shown that WHR is positively associated with male age. Interestingly, in our study, men with central obesity presented a significantly lower number of morphologically normal sperm cells and lower sperm motility and vitality than did subjects with a normal WHR (<1), which was not observed in the comparison of BMI-dependent groups. These results are consistent with findings obtained by other authors, where abdominal obesity was suspected to be a causative factor of decreased basic semen parameters. In the previously mentioned publication (see Section 3.1), Keszthelyi et al. [28] reported that WHR was significantly correlated with sperm count, motility and morphology. Notably, the authors revealed a stronger association between WHR and total sperm count and sperm progressive motility than between the semen parameters and BMI. Additionally, in one of the most recent publications, Wang et al. [13] reported that increasing WHR was linked with a reduction in semen parameters (semen volume, total number of sperm cells, total number of sperm cells with progressive motility and total number of motile spermatozoa). On the basis of the above data, it can be assumed that central obesity has a negative effect on semen quality and ultimately on male fertility.

On the other hand, some studies fail to confirm that WHR significantly influences basic semen parameters [12,27,40]. Wang et al. [40] reported that there were no significant relationships between WHR and conventional semen parameters and that there were no significant differences in the distributions of men with azoospermia, oligozoospermia, asthenozoospermia or teratozoospermia between WHR-dependent groups. Therefore, the authors concluded that semen quality is independent of WHR. Moreover, Lu et al. [27] and Esmaeili et al. [12] reported no linear associations between WHR and basic semen parameters.

Undoubtedly, these different results make it difficult to draw clear conclusions regarding the effects of BMI and WHR on basic semen parameters. On the one hand, the harmful effects of anthropometric obesity-related markers on sperm count, morphology, motility, and vitality seem to be highly probable. On the other hand, obesity is not a surrogate for low semen quality and male infertility.

### 3.3. BMI and WHR Have an Unclear Influence on Nonstandard Semen Test Results

Standard semen analysis is the first step of male fertility evaluation. However, this analysis is an insufficient tool to predict the ability of sperm cells to fertilize. Therefore, we extended basic semen analysis to assess essential parameters for fertilization and embryo development in natural and assisted reproduction [47,48].

Many authors emphasize the significant role of oxidative stress in semen in the etiopathogenesis of male infertility [49,50,51]. Additionally, in the case of obesity, the occurrence of inflammation and oxidative stress, including in the male reproductive system, is suspected [20,21,22]. To determine whether obesity is a risk factor for disrupted redox balance, we evaluated two independent variables commonly used to detect oxidative stress, namely nsORP in semen and TAC in the seminal plasma. Notably, the obtained results are unexpected. No significant differences in the nsORP or TAC values, incidence of oxidative stress in semen (nsORP > 1.37) or low total antioxidant capacity of seminal plasma (TAC ≤ 1950) were noted between men with obesity (BMI ≥ 30 kg/m^2^ and WHR ≥ 1) and men with a normal BMI and WHR. Consequently, there was no risk for oxidative stress or low TAC in the compared groups.

To the best of our knowledge, no studies have verified the relationship between WHR and oxidative stress in semen. Most authors [11,14,23,44] focus their attention on BMI and report results opposite to ours. Abbasihormozi et al. [11] demonstrated that males with obesity (BMI ≥ 30) had significantly greater amounts of ROS and lower TAC in semen than nonobese men did (BMI < 30). The authors did not find a significant correlation between BMI and ROS production but observed a very weak significant association between BMI and TAC (r = −0.19). In other studies, significant and stronger relationships between BMI and ROS production [44] or markers of seminal oxidative stress (8-hydroxy-2′-deoxyguanosine [8OHdG]), a marker of oxidative damage of DNA) [23] were found. Moreover, experimental studies using an animal model confirmed that obesity is associated with an increase in ROS generation and a decrease in the expression of enzymatic antioxidant mRNAs [14].

Considering the presented data and the results of our study, the relationship between BMI and oxidative stress in semen may not always be obvious. Abnormal body weight, without verification of oxidative stress in semen, should not be considered a definitive diagnostic criterion for the administration of antioxidant therapy. In numerous studies, antioxidant therapy is recommended for men suffering from infertility; however, the administration of antioxidant therapy in the absence of oxidative stress may cause reductive stress (diagnosed, among others, when nsORP is below –9.76 mV/10^6^ sperm cells/mL), which may also contribute to male fertility disorders. This phenomenon is called the antioxidant paradox [52,53,54].

In addition to assessing nsORP and TAC in semen, we verified the ability of sperm cells to bind with hyaluronic acid (sperm–hyaluronan-binding assay [HBA]). It has been suggested that only mature spermatozoa without chromosomal aneuploidy and sperm DNA fragmentation contain hyaluronan-binding receptors and are able to connect with hyaluronic acid/hyaluronan (HA) localized in the matrix of cumulus cells and finally reach the zona pellucida of the oocyte [55,56]. Considering the previously described harmful effects of obesity, particularly central obesity, on spermatogenesis, we investigated whether men with an abnormal BMI and WHR have significantly lower HBA index results and a greater frequency and risk for low HBA index results (<80%) than men with a normal BMI and WHR. On the basis of these data, we cannot confirm that obesity negatively influenced sperm maturity in our study subjects. No significant differences in HBA index results were noted between men who were overweight or obese and those with a normal BMI and WHR. This observation was consistent with the findings of other researchers, who noted that men with overweight and obesity do not differ in the number of sperm cells that bind to hyaluronic acid [57]. However, some authors [55] have suggested that increasing BMI could be a causative factor of male infertility because of the reduced ability of sperm cells to recognize the matrix of cumulus cells and fertilize the oocyte. Notably, the number of studies on the relationship between BMI and HBA index results is limited.

Finally, SDF was the last nonstandard semen parameter analyzed in this study. Currently, many authors have highlighted that the assessment of sperm DNA integrity is crucial for verifying the causes of male infertility. Abnormal sperm genome integrity negatively affects the fertilization process, embryo development, and chances of achieving pregnancy. Moreover, a high SDF index can cause pregnancy loss [4,30,31,32,33]. Therefore, we decided to verify the possible link between obesity and sperm chromatin integrity. Our findings revealed that men with a BMI ≥ 30 kg/m^2^ presented a significantly greater proportion of sperm cells with fragmented DNA, a greater incidence of individuals and a greater risk for clinically significant sperm DNA damage (SDF index > 20%) than men with a BMI of 25.0–29.9 kg/m^2^. Similarly, on the basis of a comparison of WHR-dependent groups, we clearly showed that central obesity was linked to higher SDF values and a higher prevalence and OR for an SDF index > 20%. These results align with other studies, which consistently demonstrated that obesity leads to pronounced negative effects on sperm DNA integrity [11,23,42,43,58]. Importantly, these findings are consistent with the results of a meta-analytic study [29] showing a significant improvement in sperm DNA integrity following weight loss. It should also be noted that, according to the most recent experimental animal studies, the obesity-related decrease in semen quality may be inherited. Li et al. [59] demonstrated the intergenerational effect of a high-fat diet and obesity-induced male infertility. The offspring of fathers with obesity presented, among other effects, a deficiency in semen antioxidants, disturbed sperm mitochondrial membrane potential, insufficient ATP content, and increased DNA fragmentation. Thus, paternal obesity can induce infertility issues in subsequent generations. The opposite results were presented in only a few reports that did not confirm the influence of male obesity on sperm DNA integrity [35,44]. Considering all the presented data, it can be assumed that the sperm chromatin status is a sensitive and independent parameter that is significantly affected by obesity.

## 4. Materials and Methods

### 4.1. Participants

The study was conducted on a general population of adult male volunteers (*n* = 543, median age: 33.00 years; range: 18.00−59.00 years) who were admitted to the Andrology Laboratory in the Department of Histology and Developmental Biology (Faculty of Health Sciences, Pomeranian Medical University in Szczecin, Szczecin, Poland). The subjects were recruited via traditional and social media advertising (2021−2024). Although the study focused on the general population, men with a medical history of cryptorchidism, testicular injuries (e.g., torsion), tumours, or orchidectomy were excluded from participation. Additionally, volunteers diagnosed with cryptozoospermia (no sperm cells in fresh semen samples but present in the centrifuged pellet) or azoospermia (no sperm cells in the ejaculate) were also excluded.

The study protocol was approved by the Ethics Committee of Pomeranian Medical University, Szczecin, Poland (ethical authorization number: KB-0012/43/2021). In accordance with the Declaration of Helsinki, all participants signed an informed consent form for participation in the research project.

### 4.2. Body Mass Index

Measurements of the participants were taken in the laboratory on the day of semen analysis. A medical scale (Charder, MS4940, Taichung, Taiwan) with a built-in telescopic height meter was used for body mass and height measurements. On the basis of the obtained data, BMI was calculated as the weight in kilograms divided by the square of the height in meters (kg/m^2^). In our study, according to the WHO recommendations, a BMI ranging from 18.5 to 24.9 kg/m^2^ indicated a normal BMI, whereas a BMI ranging from 25.0 to 29.9 kg/m^2^ indicated overweight, and a BMI ≥ 30.0 kg/m^2^ indicated obesity [https://www.who.int/news-room/fact-sheets/detail/obesity-and-overweight (accessed on 25 February 2025)].

### 4.3. Waist–Hip Ratio

Waist circumference was measured at the approximate midpoint between the lower margin of the last palpable rib and the top of the iliac crest, whereas hip circumference was measured around the widest portion of the buttocks. During the measurement, the patients stood in a relaxed posture with their arms placed at their sides, their feet placed close to each other and their body weights evenly distributed. The participants wore light clothing to minimize measurement error. The measurement was performed using a fixed tension providing tape. The waist–hip ratio (WHR) was calculated as the waist circumference divided by the hip circumference. In our study, a WHR less than 1 was considered normal, whereas a WHR greater than or equal to 1 was considered indicative of central (abdominal) obesity and was associated with an increased risk of cardiovascular and metabolic disorders [60,61].

### 4.4. Basic Semen Analysis

Following the sixth edition of the WHO guidelines [62], semen samples were collected by masturbation after 2−7 days of sexual abstinence into a wide-mouth sterile plastic graded container and stored at 37 °C. Semen analysis was performed 30 min after ejaculation using fully liquefied samples. Basic semen assessment included the following macroscopic examinations: semen volume, time of liquefaction, pH, and viscosity. Microscopic examinations included sperm concentration; motility, morphology and viability; and leukocyte concentration in semen. To determine the concentration of the sperm cells in 1 mL of semen (×10^6^ sperm cells/mL), the sample was loaded into an improved Neubauer counting chamber (Heinz Hernez Medizinalbedarf GmbH, Hamburg, Germany) and examined at a magnification of ×400 under phase contrast illumination (DM500 light/phase-contrast microscope, Lecia, Heerbrugg, Switzerland). The total sperm count in the ejaculate was calculated by multiplying the concentration of sperm cells by the semen sample volume (×10^6^ sperm cells in the ejaculate). Sperm motility was evaluated in native semen and graded as progressive motility (cells that move in a mostly straight line or in very large circles), nonprogressive motility (other types of sperm cell movement) and immotile sperm cells (complete lack of any movement) (magnification of ×400 under phase contrast illumination). Total sperm motility was calculated as the sum of spermatozoa with progressive and nonprogressive motility. To evaluate sperm cell viability, an eosin test (eosin-negative sperm cells considered live cells) and a hypo-osmotic swelling test (HOS test, hypo-osmotic-reactive sperm cells considered live cells) were used. The assessments were performed under a bright light microscope (magnification of ×400). The morphological features of the spermatozoa (Papanicolaou staining) were assessed using Tygerberg’s Strict Criteria recommended by the WHO [62] (bright light microscope, magnification of ×1000, oil immersion lens). The cells were scored as normal or abnormal. Additionally, based on number of sperm head, midpiece and tail defects as well as the number of immature sperm cells with residual cytoplasm (detailed sperm morphology evaluation), the teratozoospermia index ([TZI], a parameter reflecting multiple morphological sperm defects) was calculated. Leukocytes (peroxidase-positive cells) were identified using the Endtz test (LeucoScreen Kit, FertiPro N.V., Beernem, Belgium). To identify leukocytes, an improved Neubauer counting chamber was used (magnification of ×400 under phase contrast illumination).

### 4.5. Oxidative–Reduction Potential

The static oxidative–reduction potential (sORP) was measured according to the MiOXSYS^®^ system manufacturer’s (Aytu BioScience, Englewood, CO, USA) protocol. A fully liquefied native semen sample (30 μL) was dropped into the sample port of a disposable galvanostatic-based sensor and inserted into the analyzer. The analyzer reads the electron flux voltage from seminal redox reactions. In this system, the result is expressed in millivolts (mV). According to the manufacturer’s recommendations, the value must be normalized to the sperm concentration and is finally reported as nsORP (expressed in mV/×10^6^ sperm cells/mL). With respect to the manufacturer’s recommendations, the cutoff value of the nsORP was 1.37. The results above 1.37 indicated oxidative stress in the semen.

### 4.6. Seminal Plasma Total Antioxidant Capacity

The total antioxidant capacity (TAC) of seminal plasma was evaluated using an antioxidant assay kit and reagents (Cat# 7090011 Cayman Chemical, Ann Arbor, MI, USA). The TAC assay verified the ability of all antioxidants in seminal plasma to inhibit ABTS^®^ (2,2′-azino-di-[3-ethylbenzthiazoline sulfonate]) oxidation to ATBS^+^, and it was performed on a 96-well plate and assayed in duplicate in accordance with the methodology described by Roychoudhury et al. [63]. Briefly, according to the manufacturer’s instructions, the reaction mixture contained 150 µL of chromogen containing ABTS, 10 µL of diluted seminal plasma with assay buffer (1:9) or a suitable standard Trolox (6-hydroxy-2,5,7,8-tetramethylchroman-2-carboxylic acid, a water-soluble tocopherol analog), 10 µL of metmyoglobin, and 40 µL of H_2_O_2_. The samples were incubated with shaking for 5 min at room temperature in a horizontal plate shaker. After incubation, the suppression of the absorbance of blue–green ABTS^+^ was measured at 405 nm in a spectrophotometric microplate reader (ELx808, Bio Tek Instruments, Inc. Winooski, VT, USA). Each sample was analyzed in duplicate. The final concentration of antioxidants (expressed as µM Trolox equivalent) was established by calculating the average absorbance of each standard and sample. The total antioxidant capacity of each sample was calculated using the linear regression of the standard curve by substituting the average absorbance values for each sample into the following equation [63]:

$$TAC\left(µMTrolox\;equivalent\right)=[\frac{\left(\mathrm{Sample average absorbance}\right)-(y-intercept)}{Slope}]\times dilution\times 1000$$

It is estimated that TAC > 1950 µM Trolox equivalent is related to the normal antioxidant capacity of seminal plasma, whereas TAC ≤ 1950 µM Trolox equivalent indicates increased ROS production and/or a reduced ability of antioxidants to scavenge the formation of ROS in semen samples, which may cause oxidative stress in semen [64].

### 4.7. Sperm–Hyaluronan-Binding Assay

The hyaluronan-binding assay (HBA^®^) is an in vitro diagnostic test that verifies the ability of sperm cells to recognize and bind hyaluronic acid, the main component of the cumulus oophorus matrix [55,56]. Following the manufacturer’s (CooperSurgical, Inc.; Trumbull, CT, USA) instructions, 7–10 µL of fully liquefied semen sample was dropped onto an area near the outside edge of the assay chamber localized in the hyaluronan-coated glass slides and covered with a coverslip. After that, the chamber was incubated for 10–20 min at 20–30 °C. After incubation, at least 100 motile sperm were assessed. The sperm were divided into two categories: bound to the layer motile sperm with no progressive motility but active tail beating (HBA-positive cells) and unbound motile sperm (HBA-negative sperm cells that swim about freely). Dead and nonmotile sperm cells were not counted in this test. According to the protocol, the percentage of sperm cells binding to the hyaluronan layer was calculated as follows:

$$HBA\;index\left(\%HBA\right)=100\times \frac{bound\;motile\;sperm\;cells}{bound+unbound\;motile\;sperm\;cells}$$

According to the manufacturer, only mature spermatozoa can bind to hyaluronic acid. HBA results ≥ 80% are considered normal (mature sperm cells with physiological function).

### 4.8. Sperm DNA Fragmentation

A sperm chromatin dispersion test (SCDt) was performed to evaluate the percentage of spermatozoa with fragmented nuclear DNA (SDF index). For this purpose, a Halosperm G2 test^®^ kit (Halotech, Madrid, Spain) was used. The samples were prepared for assessment according to the manufacturer’s guidelines enclosed in the kit. The manufacturer recommends that the sperm cell concentration should not exceed 20 × 10^6^ cells/mL. If necessary, phosphate-buffered saline (PBS) should be used as a diluent. Next, the semen sample was mixed with melted agarose (in a ratio of 1:2; 50 μL:100 μL) at 37 °C. Then, 8 μL of the suspension was dropped onto a supercoated slide, which was immediately covered with a glass coverslip and incubated for 5–10 min at 4 °C. After agarose solidification, the coverslip was removed by gently sliding it off. All steps from this point on were performed at room temperature (22 °C). The reaction area on the slide was immersed (horizontally) in a ready-to-use acid denaturation solution (DA) for 7 min, after which the DA was removed by tilting until the slide was completely dry. In the next step, the reaction area was covered with a ready-to-use lysis solution (LS) for 20 min. After the incubation time, the LS was removed, similar to the DA. After drying, the slides were washed for 5 min with distilled water and then dehydrated sequentially in 70% and 100% ethanol for 2 min each. Finally, the slides were stained with ready-to-use eosin staining solution A and thiazine staining solution B for 7 min each.

SDF evaluation was performed manually under a bright light microscope (magnification of ×400, DM500 light microscope, Lecia, Heerbrugg, Switzerland). An integral sperm genome was considered to be intact if the DNA was able to generate loops (halos) visible outside of the sperm head. In SCDt, SDF-negative cells were categorized into two populations of spermatozoa: those with a halo size from >1/3 to 1 of the diameter of the core (medium halo) and those with a halo greater than the diameter of the core (large halos). Spermatozoa unable to generate at least a medium halo (spermatozoa with a small halo, no halo, or with an irregularly/weakly stained core or degraded chromatin) were scored as SDF-positive cells. According to the WHO [62] recommendations, a minimum of 500 spermatozoa per sample were assessed. The results are presented as the total number of SDF-positive cells divided by the total number of assessed sperm cells and multiplied by 100. Therefore, the SDF index denotes the percentage of sperm with damaged DNA.

According to the manufacturer’s protocol and most recent meta-analyses and scientific reports, the cutoff value of the SDF index was 20%. This threshold is most useful for distinguishing fertile from infertile men [30,31].

### 4.9. Statistical Analysis

Because normally distributed continuous data were rejected (*p* > 0.05 in the Shapiro–Wilk test), continuous variables were compared using nonparametric tests. For comparisons between two groups, the Mann–Whitney U test was performed. For comparisons among more than two groups, the Kruskal–Wallis test followed by Dunn’s multiple comparison test for post hoc analysis was applied. On the basis of the cutoff values of the nsORP, TAC, HBA index and SDF index, the percentages of subjects with nsORP > 1.37, TAC ≤ 1950, HBA index < 80% and SDF index > 20% were calculated, and the prevalence of participants in the groups was compared using the Chi^2^ test. Odds ratios (ORs) with 95% CIs (95% confidence intervals) for nsORP > 1.37, TAC ≤ 1950, HBA index < 80% and SDF index > 20% values in the study groups were calculated. A *p* value < 0.05 was considered significant for all the statistical tests. Statistical analysis was performed using Statistica version 13.3 (StatSoft, Krakow, Poland) and MedCalc version 22.009 (MedCalc Software, Ostend, Belgium).

## 5. Limitations of the Study

Some important limitations of our research should be acknowledged. Firstly, our study focused on the general population of adult men; therefore, the inclusion criteria may be considered relatively broad (see Section 4.1). However, certain health conditions (e.g., endocrine or cardiovascular disorders) can be consequences of overweight and obesity, and thus could not be used as exclusion criteria. It is also well established that factors other than overweight and obesity—such as behavioral habits—may negatively impact male fertility [43]. However, due to the open accessibility of the study to a wide group of adult men, additional risk factors (such as smoking) were not included as exclusion criteria. We assumed that the likelihood of their occurrence was independent of body weight and similarly distributed across both BMI- and WHR-dependent groups.

Additionally, the BMI- and WHR-dependent groups differed significantly in age. Men exhibiting abnormal BMI and WHR values being significantly older than those with normal measurements. This is an important consideration, as in medical research—particularly in studies on human fertility—it is highly desirable to compare groups with no statistically significant differences in age. Male aging is one of the factors that can significantly influence semen parameters, as demonstrated in our previous publications [65,66]. However, it should be also noted that unfortunately often weight gain appears to be a suspected consequence of aging, a point we acknowledged in the discussion (see Section 3.1).

Notably, the evaluation of ORP in semen using the MiOXSYS system remains the subject of ongoing scientific discussion and, at times, even controversy [67,68]. On the one hand, this diagnostic method offers significant advantages, including ease of use, rapid measurement, and a relatively low risk of analytical or interpretative error. Additionally, according to the manufacturer, it enables the detection of both known and unknown oxidants and reductants present in semen. On the other hand, the method is sensitive to semen viscosity and may not be applicable in samples with increased viscosity. Furthermore, the calculation method—dividing the measured ORP value (expressed in mV) by the sperm concentration—raises certain concerns. Specifically, this approach implies that samples with very low sperm concentration are more likely to yield results indicative of oxidative stress, whereas samples with very high sperm concentration may produce results suggesting a normal redox potential, regardless of the actual mV value [67,68,69,70]. Another controversial issue is the determination of the optimal cutoff value of the nsORP. In our study, we chose to rely on the threshold provided by the MiOXSYSY system manufacturer. However, various alternative cutoff values have been reported for this assay [e.g., 1.34; 1.36; 1.42; 1.48; 1.53; 1.57 mV/×10⁶ sperm cells/mL], which may complicate the selection of the most appropriate threshold [68,70]. At the same time, although the sixth edition of the WHO guidelines [62] acknowledges the clinical utility of ORP assessment, it does not specify a cutoff value that would differentiate fertile from infertile men.

## 6. Conclusions

On the basis of the obtained results, we can conclude that in our study population, an abnormal WHR (≥1) had a greater negative impact on standard semen parameters than an abnormal BMI (≥25.0 kg/m^2^); hence, an abnormal WHR can be considered a greater threat to standard semen quality than an abnormal BMI. This parameter appears to be a more accurate indicator than BMI for evaluating the impact obesity on semen parameters. Therefore, the anthropometric assessment of men should include WHR measurement. However, the influence of obesity on nonstandard semen parameters is unclear because abnormal BMI and WHR negatively influence only chromatin status, increasing sperm DNA fragmentation. No differences in the nsORP, TAC or HBA index results were observed. Therefore, we suggest that in our study subjects, obesity can be considered a risk factor for sperm DNA damage, which is associated with increased incidences of reproductive failure (Figure 1).

## Figures and Tables

**Table 1:** Descriptive statistics and comparisons of age and anthropometric parameters across BMI-dependent groups.

Parameters	Total (*n* = 543)	Men with BMI < 25.0 (kg/m^2^) Group 1 (*n* = 209)	Men with BMI 25.0–29.9 (kg/m^2^) Group 2 (*n* = 218)	Men with BMI ≥ 30.0 (kg/m^2^) Group 3 (*n* = 116)	*p* Group 1 vs. 2	*p* Group 1 vs. 3	*p* Group 2 vs. 3
Age (y)	33.00 18.00–59.00	32.00 18.00–59.00	33.00 20.00–56.00	34.00 22.00–50.00	0.000540	0.000081	NS
BMI (kg/m^2^)	26.12 18.50–50.25	23.30 18.50–24.97	26.89 25.00–29.90	32.25 30.00–50.25	<0.000001	<0.000001	<0.000001
WHR	*n* = 529 0.980 0.580–1.660	*n* = 204 0.94 0.58–1.42	*n* = 209 0.99 0.66–1.66	1.01 0.88–1.25	<0.000001	<0.000001	0.000134

Data are expressed as the median and range. BMI—body mass index, *n*—number of subjects, NS—not statistically significant, WHR—waist–hip ratio. Statistical significance in the Kruskal–Wallis test followed by Dunn’s multiple comparison test was noted when *p* < 0.05.

**Table 2:** Descriptive statistics and comparison of study parameters in BMI-dependent groups.

Parameters	Total (*n* = 543)	Men with BMI < 25.0 (kg/m^2^) Group 1 (*n* = 209)	Men with BMI 25.0–29.9 (kg/m^2^) Group 2 (*n* = 218)	Men with BMI ≥ 30.0 (kg/m^2^) Group 3 (*n* = 116)	*p* Group 1 vs. 2	*p* Group 1 vs. 3	*p* Group 2 vs. 3
Semen volume (mL)	3.00 0.20–9.50	3.00 0.20–8.00	3.50 0.50–9.50	3.00 0.50–8.00	NS	NS	NS
Sperm concentration (×10^6^/mL)	28.00 0.18–360.00	25.67 0.34–360.	31.21 0.40–210.00	27.12 0.18–170.00	NS	NS	NS
Total number of spermatozoa (×10^6^)	84.38 0.27–840.00	75.76 0.50–759.37	99.52 1.60–840.00	78.81 0.27–829.50	NS	NS	NS
Morphologically normal spermatozoa (%)	1.000 0.00–12.00	2.00 0.00–12.00	1.50 0.00–12.00	1.00 0.00–9.00	NS	NS	NS
Total sperm head defects (%)	*n* = 541 97.00 78.00–100.00	97.00 78.00–100.00	*n* = 216 97.00 81.00–100.00	97.00 84.00–100.00	NS	NS	NS
Total sperm midpieces defects (%)	*n* = 541 37.00 9.00–88.00	38.00 9.00–85.00	*n* = 216 37.00 9.00–88.00	35.50 11.00–78.00	NS	NS	NS
Total sperm tail defects (%)	*n* = 541 29.00 6.00–100.00	28.00 6.00–100.00	*n* = 216 29.00 7.00–88.00	29.00 12.00–73.00	NS	NS	NS
Immature sperm with excess residual cytoplasm (%)	*n* = 541 4.00 0.00–41.00	3.00 0.00–30.00	*n* = 216 4.00 0.00–23.00	4.00 0.00–41.00	NS	NS	NS
TZI	1.71 1.15–2.71	1.68 1.15–2.71	1.71 1.20–2.46	1.69 1.25–2.52	NS	NS	NS
Progressive motility (PR) (%)	51.00 0.00–94.00	51.00 0.00–94.00	54.00 0.00–87.00	48.50 1.00–85.00	NS	NS	0.037064
Nonprogressive (NP) motility (%)	6.00 0.00–34.00	6.00 0.00–33.00	*n* = 218 5.00 0.00–32.00	7.00 0.00–34.00	NS	0.020333	0.002989
Total sperm motility (PR + NP) (%)	59.00 0.00–98.00	59.00 0.00–98.00	62.00 0.00–95.00	116 56.00 5.00–96.00	NS	NS	NS
Eosin-negative spermatozoa—live cells (%)	76.00 10.00–98.00	77.00 32.00–98.00	77.00 10.00–95.00	74.00 33.00–98.00	NS	NS	NS
HOS test-positive spermatozoa—live cells (%)	*n* = 486 74.00 0.00–98.00	*n* = 181 75.00 11.00–98.00	*n* = 202 75.00 0.00–91.00	*n* = 103 71.00 34.00–96.00	NS	NS	NS
Peroxidase-positive cells (×10^6^/mL)	0.25 0.00–33.88	0.25 0.00–33.88	0.25 0.00–12.50	0.25 0.00–10.25	NS	NS	NS

Data are expressed as the median and range. BMI—body mass index, HOS test—hypo-osmotic swelling test, *n*—number of subjects, NS—not statistically significant, TZI—teratozoospermia index. Statistical significance in the Kruskal–Wallis test followed by Dunn’s multiple comparison test was noted when *p* < 0.05.

**Table 3:** Prevalence and ORs for abnormal levels of nsORP, TAC, HBA and SDF in BMI-dependent groups.

Level of Analysed Biomarkers	Total % (*n*/*N*)	Group of men with BMI < 25.0 (kg/m^2^) % (*n*/*N*)	Group of men with BMI 25.0–29.9 (kg/m^2^) % (*n*/*N*)	Group of men with BMI ≥ 30.0 (kg/m^2^) % (*n*/*N*)	*p* Group 1 vs. 2	*p* Group 1 vs. 3	*p* Group 2 vs. 3	OR1 (95% CI) *p*	OR2 (95% CI) *p*	OR3 (95% CI) *p*
nsORP > 1.37 (mV/10^6^ sperm cells/mL)	41.93 (104/248)	47.87 (45/94)	35.29 (36/102)	44.23 (23/52)	NS	NS	NS	0.5939 0.3348–1.0537 NS	0.8636 0.4372–1.7057 NS	1.4540 0.7355–2.8743 NS
TAC ≤ 1950 (μM Trolox equivalent)	48.83 (59/122)	38.09 (16/42)	53.57 (30/56)	41.66 (10/24)	NS	NS	NS	1.8750 0.8302–4.2345 NS	1.1607 0.4173–3.2284 NS	0.6190 0.2355–1.6275 NS
HBA index < 80%	47.00 (55/117	46.00 (23/50)	46.66 (21/45)	50.00 (11/22)	NS	NS	NS	1.0272 0.4580–2.3036 NS	1.1739 0.4302–3.2034 NS	1.1429 0.4119–3.1710 NS
SDF index > 20%	35.91 (195/543)	35.88 (75/209)	30.73 (67/218)	45.68 (53/116)	NS	NS	0.0068	0.7928 0.5296–1.1866 NS	1.5031 0.9471–2.3855 NS	1.8960 1.1909–3.0185 0.0070

95% CI—95% confidence interval, BMI—body mass index, HBA—hyaluronic acid-binding assay, *n*—number of subjects, *N*—size of group, NS—not statistically significant, nsORP—normalized static oxidative–reduction potential in semen, SDF—sperm DNA fragmentation, OR—odds ratio, TAC—total antioxidant capacity, WHR—waist–hip ratio. OR1—OR for nsORP, TAC, HBA and SDF above the chosen values in men with a BMI of 25.0–29.0 (kg/m^2^) vs. those with a BMI < 25.0 (kg/m^2^); OR2—OR for sORP, TAC, HBA and SDF above the chosen values in men with a BMI ≥ 30.0 vs. those with a BMI < 25.0 (kg/m^2^); OR3—OR for nsORP, TAC, HBA index and SDF index above the chosen values in men with a BMI ≥ 30.0 (kg/m^2^) vs. those with a BMI of 25.0–29.9 (kg/m^2^). Statistical significance according to the Chi^2^ test and OR test was noted when *p* < 0.05.

**Table 4:** Descriptive statistics and comparisons of age and anthropometric parameters in WHR-dependent groups.

Parameters	Total (*n* = 532)	Men with WHR < 1 (*n* = 325)	Men with WHR ≥ 1 (*n* = 207)	*p*
Age (y)	33.00 18.00–59.00	32.00 18.00–50.00	34.00 23.00–59.00	0.000006
BMI (kg/m^2^)	*n* = 529 26.10 18.50–50.25	*n* =325 25.00 18.50–50.25	*n* = 204 27.90 20.25–48.8890	< 0.000001
WHR	*n* = 529 0.980 0.580–1.660	*n* = 204 0.94 0.58–1.42	1.02 1.00–1.66	< 0.000001

Data are expressed as the median and range. BMI—body mass index, *n*—number of subjects, WHR—waist–hip ratio. Statistical significance according to the Mann–Whitney U test was noted when *p* < 0.05.

**Table 5:** Descriptive statistics and comparison of study parameters in WHR-dependent groups.

Parameters	Total (*n* = 532)	Men with WHR < 1 (*n* = 325)	Men with WHR ≥ 1 (*n* = 207)	*p*
Semen volume (mL)	3.00 0.20–9.50	3.00 0.20–9.50	3.00 0.50–8.50	NS
Sperm concentration (×10^6^/mL)	28.065 0.18–360.00	28.50 0.34–360.00	27.13 0.18–143.50	NS
Total number of spermatozoa (×10^6^)	83.475 0.27–840.00	87.60 0.50–840.00	81.25 0.27–615.00	NS
Morphologically normal spermatozoa (%)	1.50 0.00–12.00	2.00 0.00–12.00	1.00 0.00–12.00	0.000742
Total sperm head defects (%)	*n* = 531 97.00 78.00–100.00	96.00 78.00–100.00	*n* = 206 98.00 81.00–100.00	0.008209
Total sperm midpieces defects (%)	*n* = 531 37.00 9.00–88.00	38.00 9.00–85.00	*n* = 206 36.00 9.00–88.00	NS
Total sperm tail defects (%)	*n* = 531 28.00 6.00–100.00	28.00 6.00–100.00	*n* = 206 29.00 6.00–88.00	NS
Immature sperm with excess residual cytoplasm (%)	*n* = 531 4.00 0.00–41.00	3.00 0.00–30.00	*n* = 206 4.00 0.00–41.00	NS
TZI	1.71 1.15–2.71	1.71 1.21–2.71	1.68 1.15–2.52	NS
Progressive motility (PR) (%)	51.00 0.00–94.00	55.00 0.00–94.00	48.00 0.00–85.00	0.000037
Nonprogressive (NP) motility (%)	6.00 0.00–34.00	6.00 0.00–34.00	5.00 0.00–33.00	NS
Total sperm motility (PR + NP) (%)	59.00 0.00–98.00	63.00 0.00–98.00	55.00 0.00–96.00	0.000010
Eosin-negative spermatozoa—live cells (%)	76.00 10.00–98.00	78.00 15.00–98.00	74.00 10.00–98.00	0.000039
HOS test-positive spermatozoa—live cells (%)	*n* = 476 74.00 0.00–98.00	*n* = 292 76.00 0.00–98.00	*n* = 184 72.00 11.00–91.00	0.002440
Peroxidase-positive cells (×10^6^/mL)	0.25 0.00–33.88	0.25 0.00–12.50	0.25 0.00–33.88	NS

Data are expressed as the median and range. HOS test—hypo-osmotic swelling test, *n*—number of subjects, NS—not statistically significant, TZI—teratozoospermia index, WHR—waist–hip ratio. Statistical significance according to the Mann–Whitney U test was noted when *p* < 0.05.

**Table 6:** Prevalence and ORs for abnormal nsORP, TAC, HBA and SDF levels in WHR-dependent groups.

Level of Analysed Biomarkers	Total % (*n*/*N*)	Group of Men with WHR < 1 % (*n*/*N*)	Group of Men with WHR ≥ 1 % (*n*/*N*)	*p* Group 1 vs. 2	OR1 (95% CI) *p*
nsORP > 1.37 (mV/10^6^ sperm cells/mL)	42.38 (103/243)	40.25 (62/154)	46.06 (41/89)	NS	1.2675 0.7485–2.1463 NS
TAC ≤ 1950 (μM Trolox equivalent)	45.83 (55/120)	46.47 (33/71)	44.89 (22/49)	NS	0.9383 0.4517–1.9490 NS
HBA index < 80%	47.74 (53/111)	50.87 (29/57)	44.44 (24/54)	NS	0.7724 0.3660–1.6301 NS
SDF index > 20%	36.09 (192/532)	30.76 (100/325)	44.44 (92/207)	0.0014	1.8000 1.2540–2.5838 0.0014

95% CI—95% confidence interval, BMI—body mass index, HBA—hyaluronic acid-binding assay, *n*—number of subjects, *N*—size of group, NS—not statistically significant, nsORP—normalized static oxidative–reduction potential in semen, SDF—sperm DNA fragmentation, TAC—total antioxidant capacity, WHR—waist–hip ratio. ORs for the nsORP, TAC, HBA index and SDF index above the chosen values in men with WHR ≥ 1 vs. men with WHR < 1. Statistical significance according to the Chi^2^ test and OR test was noted when *p* < 0.05.

## Data Availability

The data that support the findings of this study are openly available in the Mendeley Data repository at http://doi.org/10.17632/xgbhdy78d3.1 (accessed on 26 March 2025).

## Abbreviations

8OHdG	8-hydroxy-2′-deoxyguanosine
95% CI	95% confidence interval
ABTS	2,2′-azino-di-(3-ethylbenzthiazoline sulfonate)
BMI	body mass index
DA	denaturation solution
HA	hyaluronic acid/hyaluronan
HBA	hyaluronic acid-binding assay
HOS test	hypo-osmotic swelling test
LS	lysis solution
*n*	number of subjects
*N*	size of group
NS	not statistically significant
nsORP	normalized static oxidative–reduction potential in semen
OR	odds ratio
ORP	oxidation–reduction potential
PBS	phosphate-buffered saline
ROS	reactive oxygen species
SCDt	sperm chromatin dispersion test
SDF	sperm DNA fragmentation
TAC	total antioxidant capacity
TZI	teratozoospermia index
WHO	World Health Organization
WHR	waist–hip ratio
